# A Perspective on Femtosecond Pump–Probe Spectroscopy in the Development of Future Sunscreens

**DOI:** 10.1021/acs.jpca.2c01000

**Published:** 2022-04-08

**Authors:** Abigail L. Whittock, Temitope T. Abiola, Vasilios G. Stavros

**Affiliations:** † Department of Chemistry, University of Warwick, Coventry CV4 7AL, United Kingdom; ‡ Analytical Science Centre for Doctoral Training, Senate House, University of Warwick, Coventry CV4 7AL, United Kingdom

## Abstract

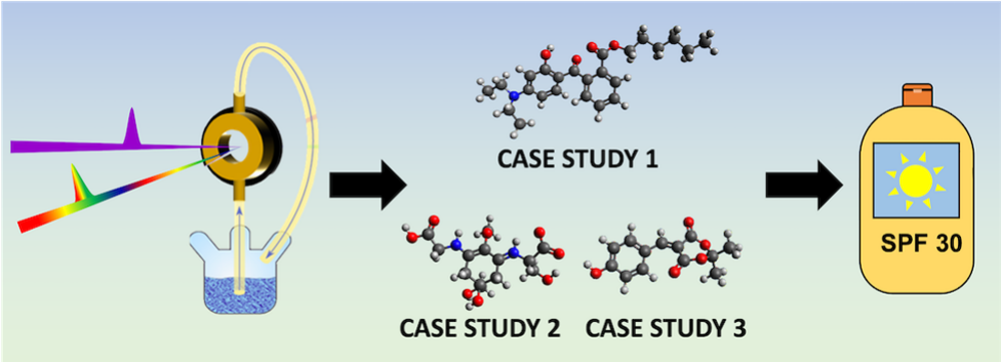

Given the negative impacts of overexposure to ultraviolet radiation (UVR) on humans, sunscreens have become a widely used product. Certain ingredients within sunscreens are responsible for photoprotection and these are known, collectively herein, as ultraviolet (UV) filters. Generally speaking, organic UV filters work by absorbing the potentially harmful UVR and dissipating this energy as harmless heat. This process happens on picosecond time scales and so femtosecond pump–probe spectroscopy (FPPS) is an ideal technique for tracking this energy conversion in real time. Coupling FPPS with complementary techniques, including steady-state spectroscopy and computational methods, can provide a detailed mechanistic picture of how UV filters provide photoprotection. As such, FPPS is crucial in aiding the future design of UV filters. This Perspective sheds light on the advancements made over the past two years on both approved and nature-inspired UV filters. Moreover, we suggest where FPPS can be further utilized within sunscreen applications for future considerations.

## INTRODUCTION

Ultraviolet radiation (UVR) makes up around 10% of the radiation emitted by the Sun prior to it entering the Earth’s atmosphere.^1^ UVR can be split into three regions; ultraviolet (UV)A (400–315 nm), UVB (315–280 nm), and UVC (280–100 nm).^2^ The ozone layer absorbs all UVC and a large proportion of UVB such that, at the Earth’s surface, only UVA and UVB radiation are present.^3^ Overexposure to UVR can lead to a number of negative effects such as skin cancer, DNA mutations, cataract formation, and photoaging to name a few.^4−12^ Further to this, depletion of the ozone layer over the last few decades has resulted in increasing levels of UVR reaching the Earth’s surface.^13^

Protection to humans from overexposure to UVR is achieved naturally through the production of melanin pigment, which is induced by UVR. This process can be supplemented by keeping UVR exposure to a minimum or through wearing protective clothing. We note, however, that melanin pigment production is a delayed and potentially inefficient process.^10,14^ Immediate protection from UVR can be achieved using sunscreens. Sunscreens have become a key cosmetic in the modern world due to the popularity of exercising and relaxing in the Sun while also offering protection from the potentially damaging effects of UVR.^14,15^ Sunscreens contain UV filters that work either by reflecting UVR or by absorbing UVR and dissipating the energy via various photophysical processes. Reflection/scattering is primarily caused by inorganic UV filters (also known as physical filters) such as titanium dioxide (worth noting that they also absorb UVR) and absorption is primarily caused by organic UV filters (also known as chemical filters) such as avobenzone.^16−18^ This Perspective will only focus on organic UV filters.

Of the approved UV filters used currently, there are several drawbacks, these being photoinstability,^19^ detrimental environmental impacts such as coral bleaching,^20,21^ and toxicity concerns to humans.^22,23^ In addition to this, there are a lack of approved UVA filters.^24^ When referring to photoinstability, a few studies have previously defined a photounstable formulation to be one in which the area under curve index for UVA and UVB regions was <0.8 after 120 min of irradiation (calculated by the area under the curve after irradiation divided by the area under the curve before irradiation).^25,26^ As a result of these drawbacks, sunscreen development is of imminent importance. One way this can be achieved is by enhancing our fundamental understanding of how UV filters dissipate the absorbed UVR. Such an understanding can be used to guide sunscreen research and development.

An ideal UV filter dissipates the absorbed UVR energy safely and rapidly, returning to its original state, via nonradiative decay pathways and on the femtosecond (10^–15^ s) to picosecond (10^–12^ s) time scale.^27^ These properties ensure that the probability of alternative reaction pathways leading, e.g., to photoproducts is minimized. Femtosecond pump–probe spectroscopy (FPPS), described in more detail within the Experimental Techniques section, can monitor the relaxation processes that molecules undergo in real time (femtoseconds, picoseconds, and nanoseconds, 10^–9^ s). It is an invaluable tool that has the ability to enlighten the sunscreen community on why a molecule would be a suitable UV filter or not. Further to this, the ideal UV filter would not penetrate the skin barrier (i.e., remain on the epidermis, outermost layer of the skin) or be toxic in any way to both humans and the environment. As a result, any candidate molecules identified through FPPS would benefit from monitoring biological end points and toxicology studies. For example, it is conceivable that a small population trapped in a low-lying triplet state of the UV filter may go undetected in FPPS measurements. This could subsequently lead to singlet oxygen being generated, which is cytotoxic and may result in skin irritation.^28,29^ Furthermore, this would be a particular problem if the UV filter was able to penetrate the skin barrier as the generated singlet oxygen could damage DNA.^30^ A recent study by Harada et al.^31^ investigated singlet oxygen’s ability to penetrate a polymer film with oxygen permeability like skin and found it to be incapable. This study reinforces the importance of designing UV filters that do not penetrate the skin barrier. One example of how this can be achieved is by designing UV filters with large molecular size. While studies involving interaction of sunscreens with the skin surface are beyond the scope of this Perspective, they highlight the multidisciplinary approach that would be required in the development of next generation UV filters.

Three case studies from the past two years are discussed within this Perspective, and we reflect on their contribution toward the advancement of sunscreen science. The first case study explores the effects of solvent on the photodynamics of an approved UV filter.^32^ The second case study examines two natural UV filters from a microbial family of photoprotective molecules.^33^ The third case study investigates plant-based UV filters in a closer-to-real-life environment including in emollient and on a synthetic skin mimic.^34^ In all case studies, we will primarily focus on the FPPS results. However, it is important to note that the selected studies employed several other techniques such as steady-state spectroscopy and computational methods. All these techniques complement one another and highlight the multitechnique approach required to advance sunscreen development.

## EXPERIMENTAL TECHNIQUES

**Figure 1:**
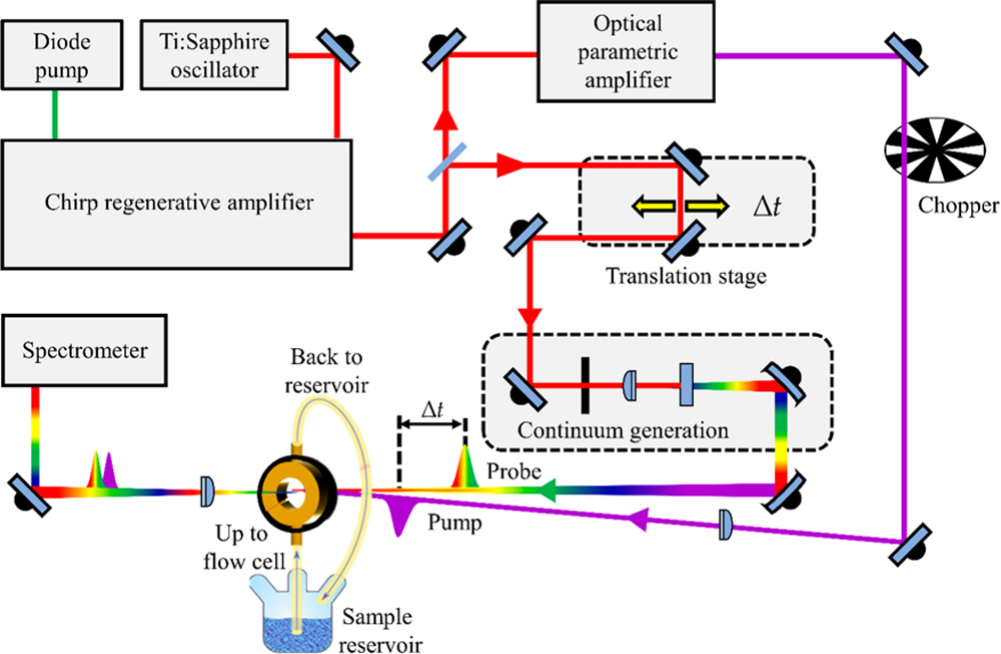
Schematic of a typical transient electronic absorption spectroscopy (TEAS) setup. Reprinted with permission from ref (38). Copyright 2020 MDPI.

Many of the photophysical and photochemical processes that accompany light absorption in UV filters occur on ultrafast time scales, i.e., within a few femtoseconds to nanoseconds. Hence, to fully understand how these dynamical processes influence the efficiency of UV filters in a sunscreen formulation, spectroscopic techniques that can resolve these ultrafast processes must be employed.^35,36^ An example of such a technique is FPPS (Figure 1), introduced *supra*, which can be employed to study molecular dynamics in both solution-phase and gas-phase environments. In this Perspective, we focus on solution-phase measurements since they are a closer mimic to the sunscreen formulation environment as opposed to isolated gas-phase measurements which provides little information about environmental perturbation.

In pump–probe spectroscopy, two laser pulses are employed to garner dynamical information from the sample of interest. The pump pulse initiates the photochemical process by exciting a portion of the sample from the electronic ground state (S_0_) to an accessible electronic excited state (S_*n*_). Thereafter, the probe pulse interacts with the already excited sample to track the excited state population at a discrete time delay (Δ*t*) relative to the initial pump pulse. By varying the Δ*t* over a long time window (femtoseconds to nanoseconds) in relatively small steps, usually tens of femtoseconds, information about the energy relaxation pathways can be obtained. A widely used pump–probe technique in sunscreen science is transient absorption spectroscopy, where changes in absorbance after pump pulse excitation are recorded with a spectrally broad probe pulse (see below) over time. The changes in the absorbance intensity of the probe wavelengths over time can display transient species such as ground state bleach (GSB), stimulated emission, excited state absorption, and absorption by any photoproduct formed. Detailed explanations of these processes can be found elsewhere.^27,37,38^ In sunscreen science, the lifetimes of the electronically excited state species are of major concern;^39^ hence the excitation pulse is usually in the UV region where photoprotection is required. Depending on the photochemical or photophysical information required, the electronically excited state can then be probed either with broad-band UV/visible wavelengths as in the case of transient electronic absorption spectroscopy (TEAS) or with infrared (IR) wavelengths as in the case of transient vibrational absorption spectroscopy (TVAS).

As shown in Figure 1, the TEAS setup comprises an ultrafast laser source, light conversion system, probe pulse delay stage, continuum (white light) generation, sample delivery system, and probe pulse detection. The tunable pump pulse wavelengths, ranging from 250 to 1000 nm are generated through an optical parametric amplifier (OPA), which allows for selective electronic excitation of the sample. The UV/visible probe pulse on the other hand is enabled by broad-band continuum generation through various media such as CaF_2_, sapphire or water. The probe pulse delay stage employs a retroreflector or a pair of broad-band mirrors mounted on a motorized translation stage to generate the pump–probe time delay (Δ*t*). The sample delivery systems range from a simple static cell, such as a cuvette, to liquid jets, and more frequently used, a flow-through cell that enables a fresh sample to be present for each laser pulse pair. Furthermore, recent advancements in sunscreen studies have presented the opportunity to deposit and probe samples on a surface, such as skin mimics or within a thin film.^34,40,41^ The probe intensities after interaction with the sample at different time delays are recorded by spectrograph combined with a silicon-based array detector, such as a charge-couple device (CCD). A mechanical chopper placed in the pump pulse path and operated at half the repetition rate of the laser source allows the probe pulse arriving at the sample to view pumped and then unpumped sample sequentially. Calculating the difference between the pumped and unpumped probe pulse gives the changes in absorbance, commonly reported as a change in optical density (ΔOD).

The TVAS setup employs the same scheme used in the TEAS, but instead of a broad-band continuum probe pulse, another OPA is required to generate IR probe pulses generally in the mid-IR region 2500–8000 nm. Also, before arrival of the probe pulse at the sample compartment, it travels through a CaF_2_ pulse splitter, which splits it equally into reference and probe pulses. The reference pulse misses the sample entirely and is detected for the subtraction of shot-to-shot laser noise. To avoid absorption of atmospheric CO_2_ and H_2_O, the entire IR probe beamline must be enclosed and purged with suitable purge gases. Contrary to the use of a silicon-based CCD detector commonly used in the TEAS setup, the IR probe pulse detection following interaction with the sample is achieved using, as a common example, a mercury cadmium telluride detector array. The detector is cooled using liquid nitrogen to reduce thermal contributions to the signal.

Several other techniques such as femtosecond stimulated Raman spectroscopy^42,43^ and time-resolved fluorescence with setups using optical Kerr gating^44,45^ or up-conversion^46,47^ are also available to complement the TEAS and TVAS measurements. In some cases, the pump–probe gas-phase measurement may be used to uncover the photofragment/photoion detection and time-resolved fluorescence.^48,49^ While the gas-phase experiments give fundamental insight into photodegradation pathways of sunscreen molecules, important information about environmental perturbation is limited and as such we reiterate that we have elected not to present any gas-phase results here.

We now turn our attention to various examples of UV filters that either are currently in use or are being developed for sunscreen formulation providing spectral coverage across both UVA and UVB regions of the electromagnetic spectrum, many of which have been studied using TEAS and TVAS.

### Case Study 1

**Figure 2:**
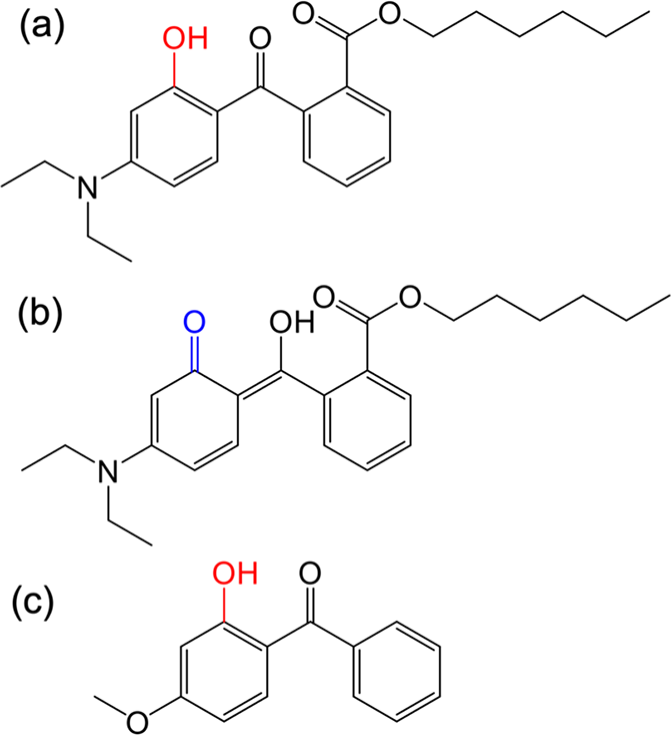
Molecular structure of (a) DHHB in its enol form, (b) DHHB in its keto form, and (c) oxybenzone in its enol form.

Diethylamino hydroxybenzoyl hexyl benzoate (DHHB), with the commercial name Uvinul A, is a widely approved UVA filter currently used in sunscreen formulations in Europe, Japan, Australia, and South Africa. DHHB has been the subject of both steady-state and FPPS studies.^18,32^ DHHB has a molecular structure similar to that of oxybenzone (see Figure 2), a widely used UVB filter with the main difference being the addition of two auxochromes to the oxybenzone core structure. The first is an amino group at a meta position to the −OH group on one phenyl ring, while the second is an ester group positioned on the other aromatic ring. These auxochromes red-shift the main π* ← π absorption band by ∼15 nm in cyclohexane solution compared to the case for oxybenzone.^32^ Previous TEAS and TVAS studies on oxybenzone revealed that the relaxation pathway is through enol → keto tautomerization mediated by intramolecular excited state hydrogen transfer (ESHT). This is followed by the central C–C bond twisting, which drives the excited state population through the S_1_/S_0_ conical intersection (CI) with subsequent vibrational energy transfer in the ground state to re-form the original enol tautomer.^50^

**Figure 3:**
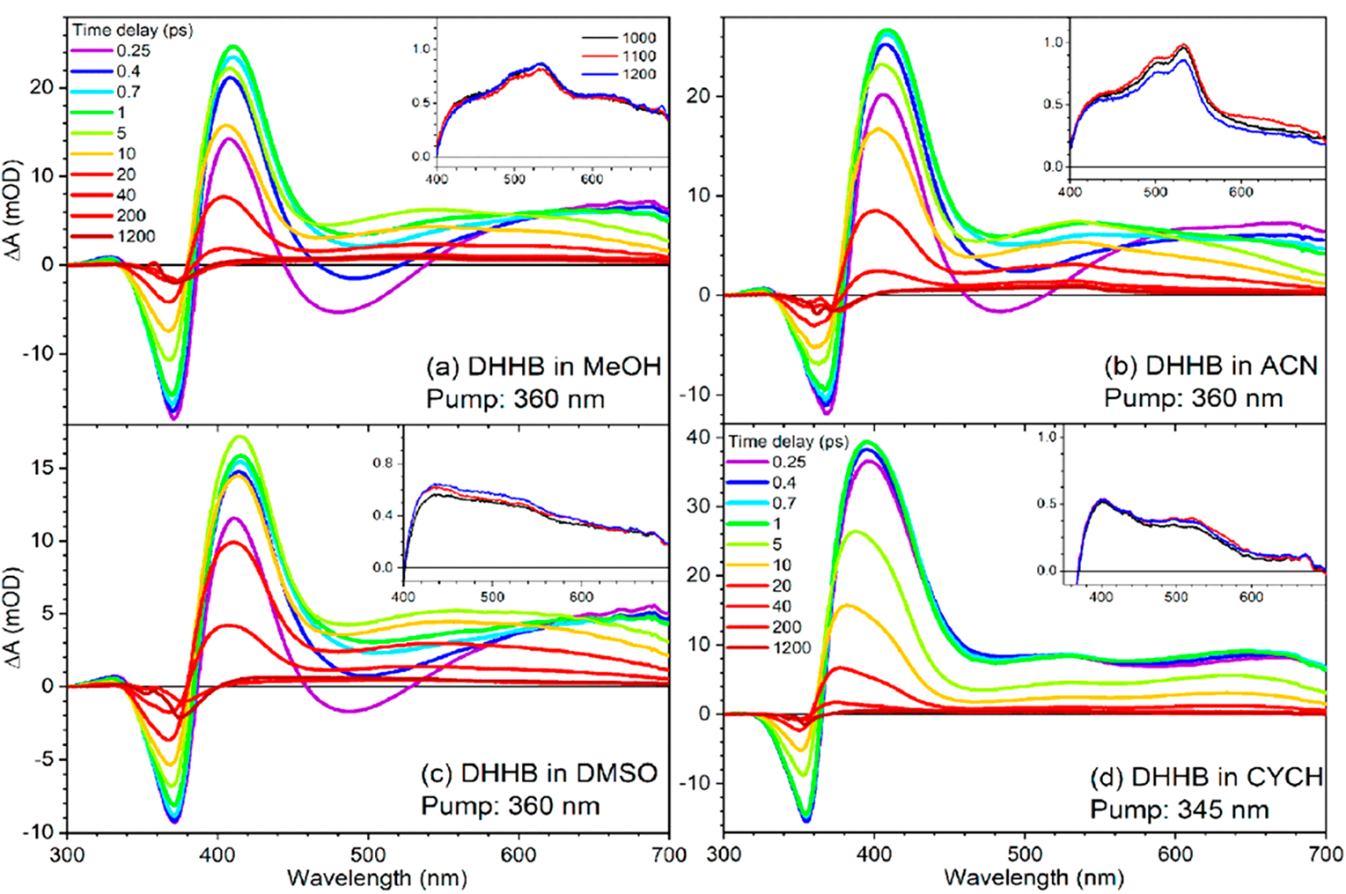
Transient electronic absorption spectra of DHHB in (a) methanol (MeOH), (b) acetonitrile (ACN), (c) dimethyl sulfoxide (DMSO), and (d) cyclohexane (CYCH) solutions. The color code indicates the spectra obtained at different pump–probe time delays. The insets in each panel show longer time delay spectra corresponding to the triplet states. Reproduced with permission from ref (32). Copyright 2021 American Chemical Society.

**Figure 4:**
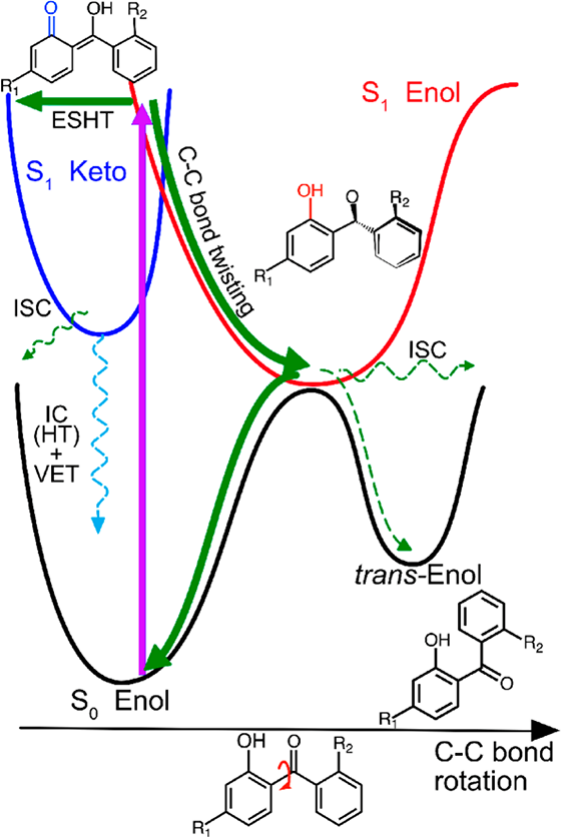
Schematic of the PES for relaxation of DHHB along the ESHT and C–C bond torsion from the S_1_ state following UV photoexcitation. Reprinted with permission from ref (32). Copyright 2021 American Chemical Society.

Recently, Kao et al.^32^ performed TEAS and TVAS measurements on DHHB to understand its photochemical properties and primary relaxation mechanism in a series of solvents, both nonpolar (cyclohexane) and polar (methanol, acetonitrile, and dimethyl sulfoxide) environments with the resulting transient electronic absorption spectra displayed in Figure 3. The authors reported that exciting DHHB at 360 nm in polar solvents and at 345 nm in the nonpolar solvent, cyclohexane, populates the first singlet excited state (S_1_) with a ππ* transition in its enol geometry in the Franck–Condon (FC) region, resulting in competing relaxation pathways that are solvent dependent. This is illustrated schematically in Figure 4. The electronic structure calculations revealed that in the S_0_ state, the carbonyl group between the two benzene rings is close to planar with the hydroxyl group having a (HO)C—C—C=O dihedral angle of 7.2°. The two phenyl rings are twisted out-of-plane by ∼47° to reduce steric repulsions.

In cyclohexane, the majority of the photoexcited DHHB molecules undergo ESHT in the FC region of the S_1_ state, converting the enol geometry to keto form within 200 fs; this is the start of the main relaxation pathway. The S_1_ keto form may then relax to the ground state via direct internal conversion (IC) and vibrational energy transfer or via reverse ESHT to repopulate the S_1_ enol form before S_1_ → S_0_ relaxation. A minor fraction of the photoexcited population, however, remains in the S_1_, which would then undergo either C−C bond torsion or intersystem crossing to the triplet state. On the other hand, in polar solvents, ESHT from the photoexcited population in the FC region is inhibited by the disruption of intramolecular hydrogen bonding in DHHB. In this case, the experimentally observed stimulated emission in the TEAS measurements is quenched competitively by the now slower ESHT and the C—C bond torsion with a solvent-dependent time constant between ∼300 and ∼800 fs. In polar solvent environments, the torsional rotation along the C–C bond brings the excited state enol tautomer of DHHB on the S_1_ potential energy surface (PES) to a CI with the electronic ground state (S_0_), denoted S_1_/S_0_ CI. The electronic structure calculations found that the optimized geometry of the S_1_ enol no longer had the carbonyl group located between the two benzene rings in the same plane as the hydroxyl group, with a (HO)C—C—C=O dihedral angle of 67° (compared to 7.2° in the S_0_ state). Furthermore, the two phenyl rings are now near perpendicular to one another. The authors also reported that the excited molecular orbital of the S_1_ enol optimized geometry shows charge transfer character and will be stabilized by polar solvents. The relaxation via this CI represents the second relaxation pathway (the first being ESHT). From this CI, the twisted enol tautomer can relax back to the starting electronic ground state enol form or produce a *trans*-enol isomer photoproduct in the electronic ground state. Since the rate of ESHT and C–C bond torsion is dependent on the solvent environment, the electronic excited state relaxation pathways of DHHB are sensitive to the solvent properties. Overall, the time scale reported for the decay of the S_1_ electronic excited state absorption band assigned to either the enol or keto tautomer ranges from 7 to 23 ps depending on the polarity of the solvent environment.

A solvent-dependent GSB recovery is also observed in the TVAS measurements, with >98% of the DHHB photoexcited to S_1_ relaxing back to the electronic ground state in cyclohexane having a time constant of ∼12 ps. The remaining <2% population in the S_1_ state in the keto form undergoes intersystem crossing to the first triplet state (T_1_) in the keto form. By comparison, in polar solvents, 95% of the photoexcited DHHB returns to the electronic ground state with a longer time constant of ∼15 ps. The remaining 5% undergo intersystem crossing from the S_1_ state in the enol form to the T_1_ keto form. To add, in a polar solvent, a small competing photoproduct, *trans*-enol isomer of DHHB, is formed by the continuing torsional motion of the C–C bond of the S_1_-enol geometry after traversing through the S_1_/S_0_ CI.

Studies such as discussed above could influence the choice of formulation environments; for example, DHHB would appear to be a better UV filter in a nonpolar environment given the higher percentage recovery of the ground state population on a shorter time scale and the absence of photoproducts formation. Taken together, this study demonstrates the importance of both TEAS and TVAS in understanding the ultrafast relaxation pathways of UV filters for sunscreen application as well as the importance of the solvent environment in their studies. Also, the work highlights the need to study UV filters in a solvent environment such as an emollient used in industry as their properties could influence the overall performance of the sunscreen.

### Case Study 2

**Figure 5:**
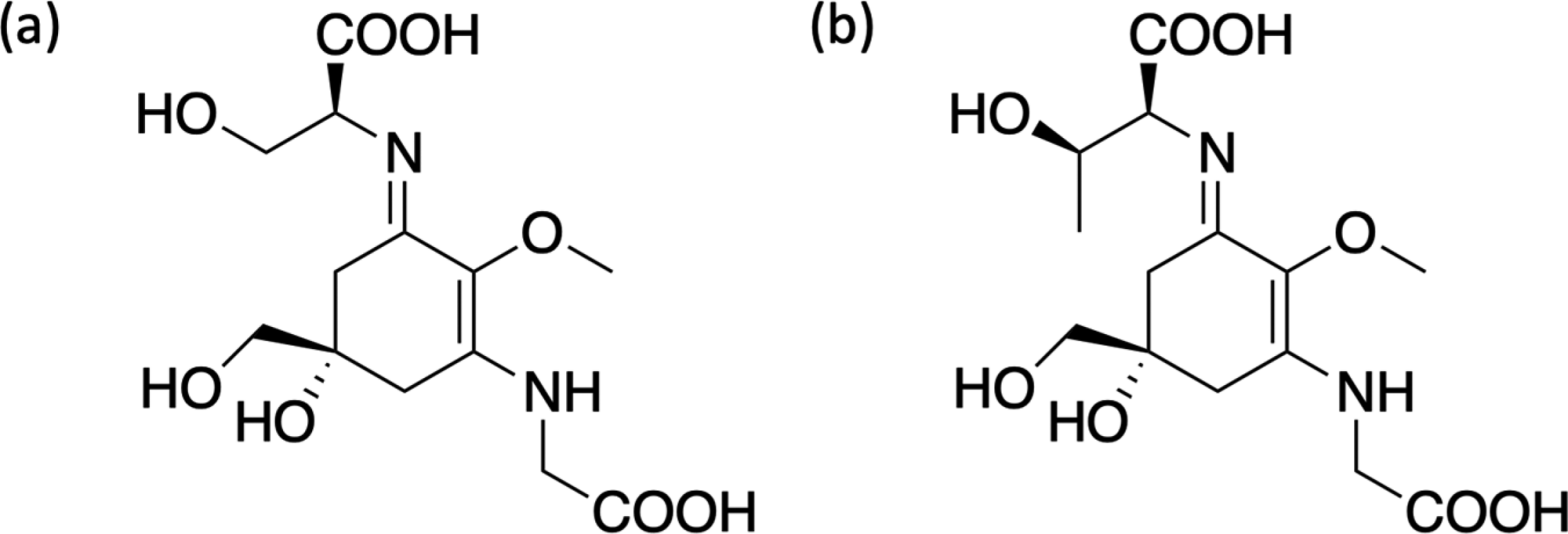
Structure of (a) shinorine and (b) porphyra-334.

Nature-inspired UV filters have been a topic of interest recently as they are suspected to overcome several drawbacks, including damaging environmental impacts and toxicity concerns to humans, as discussed in the Introduction.^38,51,52^ Natural organisms produce various photoprotective families. One of these families is mycosporine-like amino acids (MAAs), which are synthesized by cyanobacteria, fungi, and algae and are believed to have multifunctionality including photoprotective and antioxidant properties.^53^ In the chosen case study, Whittock et al.^33^ investigated two MAAs, shinorine and porphyra-334, using FPPS, steady-state spectroscopy and computational methods; structures for these two molecules are given in Figure 5. This study is believed to be the first FPPS on natural MAAs and as such has provided new insight into the relaxation dynamics of these molecules. It is worth noting that prior to this study on natural MAAs, Losantos et al.^54^ and Woolley et al.^55^ studied synthetic MAA derivatives using TEAS. Both studies showed promising results and guided the analysis within the present case study as well as demonstrated how different substituents influence the photoprotective mechanism.

**Figure 6:**
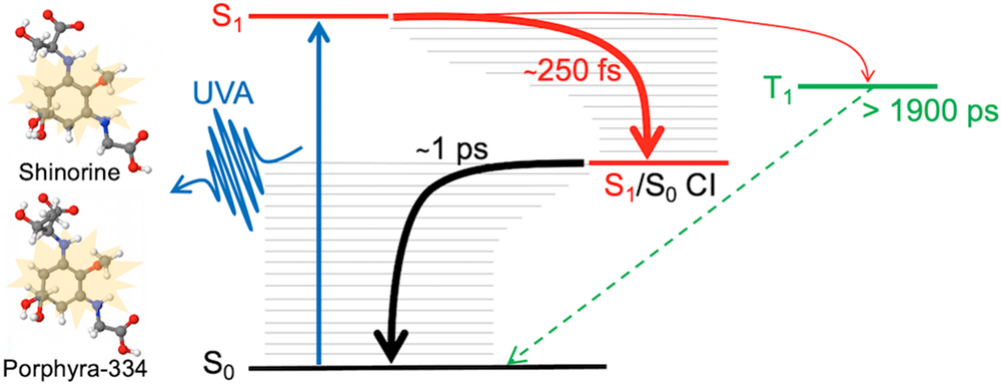
Schematic of the photoprotection mechanism of the MAAs shinorine and porphyra-334. Reprinted with permission from ref (33). Copyright 2021 American Chemical Society.

Shinorine and porphyra-334 have a strong UVA absorption around 330 nm assigned to an S_1_ ← S_0_ transition with ππ* character. The authors determined that following photoexcitation to this first electronic excited state (S_1_), these MAAs relax along the S_1_ PES toward an energetically accessible CI with an assigned lifetime of a few hundred femtoseconds. Drawing upon previous high-level computational studies, the mechanism they proposed was via a planar to nonplanar ring flexing motion whereby one of the amino acid arms folds out of the plane of the ring.^56−59^ Following this, the population traverses through the CI to populate the vibrationally hot electronic ground state where it subsequently cools; combined, these processes occur within ∼1 ps. The fast vibrational cooling was proposed to be due to the large hydrogen bonding network to the solvent, which is further strengthened by the zwitterionic nature of MAAs.^60^ A schematic of the photoprotective mechanism is depicted in Figure 6 below.

**Figure 7:**
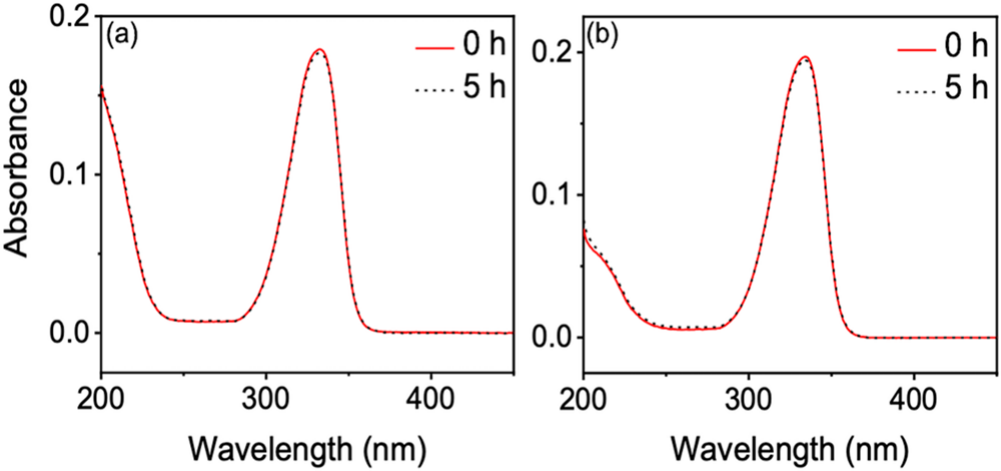
UV–visible spectra before and after 5 h of irradiation with a solar simulator for (a) shinorine and (b) porphyra-334. Reprinted with permission from ref (33). Copyright 2021 American Chemical Society.

Through assessment of the GSB recovery at the maximum time delay, Δ*t* = 1900 ps, a minor portion (≤5%) of the population appears to follow a different pathway with a lifetime of >1900 ps. The authors suggest this could be the result of three processes, photoproduct formation, or trapped population in either the singlet or triplet state. By using previous literature on the fluorescence and triplet quantum yields,^61,62^ in addition to their calculations and steady-state spectroscopy, the authors proposed that the most likely contributing factor is trapped population in the triplet state.^33^ Furthermore, upon irradiating samples for 5 h with a solar simulator (relevant to sunscreens as output power is equivalent to the sun at the Earth’s surface), very little change in the UV–visible spectra was observed, ∼1%, as can be observed in Figure 7, reinforcing the high level of UVR that MAAs can withstand. Within the present case study, the authors believe that the triplet state likely finds a way back to its original ground state beyond 1900 ps given the steady-state irradiation results. However, we must add that in a more complex mixture such as a sunscreen formulation, this triplet state may pose a threat as it could lead to singlet oxygen generation which, as we discussed in the Introduction, could possibly lead to skin irritation and DNA damage. We note that in a review by Singh et al.,^63^ the ability of MAAs to protect the skin from UV damage and toxicity studies are summarized with promising results. However, there is certainly scope to extend the knowledge of MAAs triplet state and toxicological properties.

The dominant photoprotective mechanism is quick and efficient demonstrating how well nature has evolved to protect itself from UVR. As MAAs were believed to be some of the earliest UV screening compounds to exist, they would have been exposed to harsher UV conditions and this could explain their well-adapted resistance to degradation upon UVR exposure.^64,65^ As such, we should continue to utilize nature’s own knowledge to further our research within the framework of the sunscreen industry. Specifically, given the long-term existence of MAAs and other natural sunscreens, it stands to reason that we should use insight from them when developing next generation UV filters. Note that, as discussed briefly above, work on synthetic derivatives of MAAs has already begun.^54,55,66^ However, we add that this extends beyond MAAs and to all of nature’s sunscreens; in Case Study 3 we will explore some plant-based UV filters in a closer-to-real-life environment.

### Case Study 3

Recently, researchers working on sunscreens have studied UV filters in a closer-to-real-life sunscreen formulation and application environment, i.e., in emollient and on a skin mimic surface. In doing so, vital information regarding the influence of formulation environments (i.e., solvents) as reported in Case Study 1, and any changes in the dynamics mediated by the skin surface on UV filters can be obtained. Such studies are important in the design of next generation UV filters to provide efficient and safe photoprotection to humans. Liu et al.^40^ were among the first to report the effects of applying a UV filter, specifically plant-inspired UV filters based on sinapate esters, to a surface mimicking skin. These sinapate esters were mixed into a poly(vinyl) alcohol (PVA) hydrogel film that the authors employed as the model skin mimic. The authors reported a 25-fold increase in the time taken for the deactivation of the electronic excited state through *trans–cis* photoisomerization compared to the values extracted from buffer solution. Following this, Horbury et al.^41^ compared the photodynamics of symmetrically substituted diethyl sinapate (DES) in different environments including emollient (C12–15 alkyl benzoate), synthetic skin mimic, VITRO–CORNEUM (VC), and in conventional solvents such as ethanol and cyclohexane. The authors reported a 3-fold increase in the deactivation lifetime of the electronic excited state for DES on the skin mimic compared to the lifetime when it is dissolved in emollient, which itself presented a much longer time than those in conventional solvents. The results of these two studies have been reviewed in detail in previous publications, and so, for Case Study 3, we move on to discuss a more recent study that builds on this work.^27,38^

**Figure 8:**
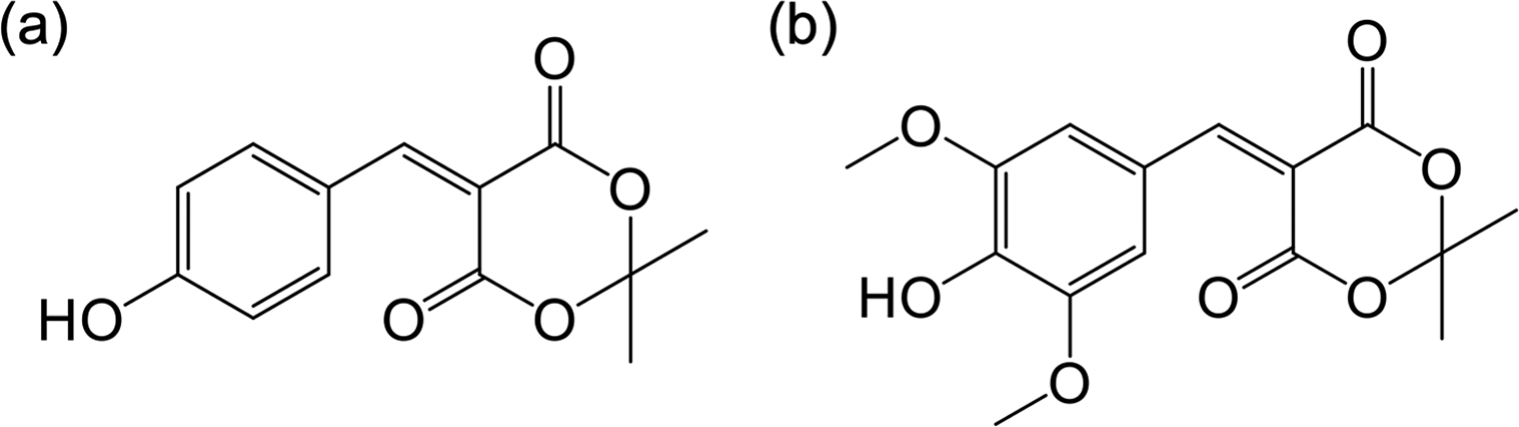
Structure of (a) coumaryl Meldrum and (b) sinapyl Meldrum.

Abiola et al.^34^ employed a multiprong approach using TEAS, TVAS, computation, and steady-state methods to unravel the photodynamics of two more symmetrically substituted plant-based UV filters, coumaryl Meldrum (CMe) and sinapyl Meldrum (SMe) shown in Figure 8. The study was carried out in industry standard emollient, caprylic capric triglyceride (CCT), on a skin mimic (VC), and in ethanol. Both Meldrums in CCT absorbed strongly in the UVA with λ_max_ of 362 and 396 nm for CMe and SMe, respectively, which corresponds to the S_1_ ← S_0_ transition with ππ* character. In ethanol, the λ_max_ is red-shifted by 10 nm in both cases, this shift to longer wavelength in a polar solvent is typical for ππ* transitions.^32,67^ The authors reported that following photoexcitation to their respective S_1_, similar relaxation pathways were observed for both Meldrums and in all studied environments (when in bulk ethanol or CCT or when deposited on VC).

**Figure 9:**
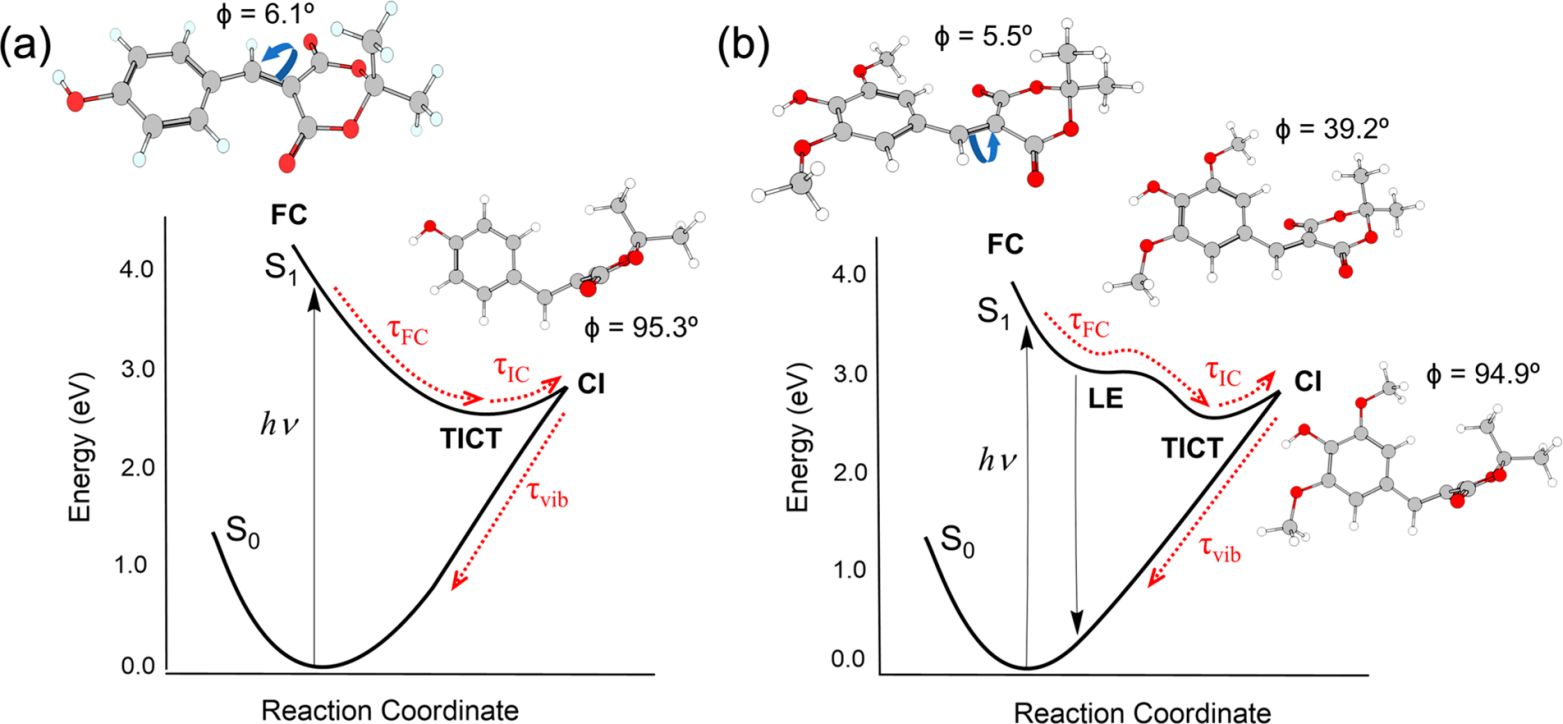
Schematic of the PES for relaxation of (a) CMe and (b) SMe. Reprinted with permission from ref (34). Copyright 2020 The Authors.

The electronic excited state deactivation mechanism involved relaxation out of the FC region into a global minimum with a ∼90° geometry twisted around the C=C allylic bond with a charge transfer character (determined through calculation) prior to reaching the S_1_/S_0_ CI. As shown in Figure 9, prior to reaching the twisted intermolecular charge transfer (TICT) global minimum, SMe relaxes through a locally excited minimum on the S_1_ PES, which is absent in the CMe PES. The authors reported that this local minimum in SMe presents a small energy barrier that must be overcome before SMe relaxes into the TICT minimum, consequently resulting in a difference in one of extracted lifetime of SMe and CMe in the femtoseconds regime. From the TICT, both CMe and SMe undergo IC through the S_1_/S_0_ CI to populate the vibrationally hot electronic ground state. Both the TICT and IC occur within a few hundred femtoseconds. The vibrationally hot electronic ground state population then transfers the excess energy to its surrounding via vibrational cooling on a time scale of 10 ps.

The reported TVAS measurements confirmed that the excess energy in CMe and SMe, following UV photoexcitation, is indeed transferred to the surrounding bath as heat. Finally, the authors suggested through calculation and steady-state measurements that the mild incomplete GSB recovery in both TEAS and TVAS measurements is unlikely to be a photoproduct but corresponds to the trapped population in the electronic excited state.

Importantly, unlike the studies by Liu et al.^40^ and Horbury et al.^41^ where the electronic excited state deactivation lifetime of the UV filters is significantly slowed down on the skin surface, Abiola et al.^34^ reported that the electronic excited state deactivation lifetime of CMe and SMe is not influenced by environment, with no significant change in the lifetime of data obtained on the skin mimic. The implication of slower relaxation of UV filters on skin is that it could give rise to (competing) harmful side reactions. Hence, sunscreen scientists should continue to study UV filters in a close-to-real-life formulation and application environment to ensure only molecules whose dynamics are not significantly influenced by these factors are further pursued.

## FUTURE WORK

In addition to the presented case studies, we briefly discuss a few avenues of further research that can be pursued for sunscreen advancement. The first involves obtaining a more in-depth understanding of structure–function properties in relation to photoprotective efficiency. This could be achieved by modifying the structures of approved and candidate UV filters and evaluating the GSB recovery; note that an ideal scenario for a UV filter would be complete recovery (assuming no convolution of positive spectral features overlapping the GSB, which could result in a false GSB recovery). Recently Holt et al.^68^ built on previous work^69^ and linked the ultrafast photophysics of avobenzone, a common UVA filter, to its long-term photostability in more complex mixtures (multiple UV filters including avobenzone in industry standard emollient). Extensive incomplete GSB recovery was observed in the authors’ transient absorption spectra of avobenzone, and unsurprisingly, they observed a SPF and UVA-PF percentage decrease upon irradiation of the more complex mixtures detailed above. This suggests that the incomplete GSB was the result of photoproducts being formed which, in turn, decreased the photoprotective efficiency of the UVA filter, as reflected in the SPF and UVA-PF percentage decrease results. With this in mind, future efforts into modifying the structure of avobenzone and monitoring the extent of GSB recovery could result in less photodegradation and greater photoprotective efficiency.

In Case Study 3, we presented studies that applied UV filters to skin mimics or incorporated them in a thin film and assessed their photodynamics in comparison to studies when the UV filter was in a solvent, one example of these skin mimics being VC.^34,40,41^ While VC models the properties of the very outer layer of the skin and enables insight into how a UV filter behaves on a surface, it does not account for the effects of oils, sweat, temperature, and other factors that may influence reactions of the UV filters with the skin. Hence, to fully account for the effects of the skin properties on a UV filters efficiency, further development of the skin mimic or changes in sample preparation might be required to incorporate all the factors that could influence photochemical processes. As this will involve increasing the complexity of the environment, we can predict that the transparency of the studied systems will decrease. These more opaque environments will prove difficult to execute transient absorption spectroscopy measurements based on light transmission through the sample. As such, we suggest that an avenue of future work is a technological advancement to the current state-of-the-art FPPS measurements with respect to sunscreen science research; this being the use of transient reflection spectroscopy. Previous literature has transformed transient reflection spectroscopy into transient absorption spectroscopy based on Kramers–Kronig relations.^70,71^ If the same approach could be taken for UV filters in these opaque environments, insight into the absorption properties of the studied UV filter could be resolved from transient reflection spectroscopy.

## CONCLUDING REMARKS

In summary, we have examined three case studies and related them to the future development of sunscreen research. In the first case study, we highlighted the importance of investigating how the solvent environment influences the observed photodynamics and how we can use this insight to optimize the formulation environment for sunscreen efficacy. In the second case study, we demonstrated the important tool of using nature as the inspiration for the UV active chromophores, while changing functional groups toward optimum sunscreen properties. In the third case study, we have shown that studying promising sunscreen candidates in a closer-to-real-life environment is of crucial importance to assess whether such environments alter the observed photodynamics.

Taken altogether, there are many roles for FPPS in the development of sunscreens. We hope that we have conveyed many avenues already being employed by research groups and areas for future work. While FPPS can provide insight into the photoprotective efficiency of a candidate UV filter, no information can be garnered on the toxicological properties. In cosmetics, this is certainly of importance and so toxicological studies on promising UV filters is a welcomed avenue of complementary research. Furthermore, studies into the long-term environmental impacts of molecules that do not degrade after extended periods of time would also be of interest.

## ABBREVIATIONS

UVR: ultraviolet radiation
UV: ultraviolet
FPPS: femtosecond pump–probe spectroscopy
GSB: ground state bleach
TEAS: transient electronic absorption spectroscopy
IR: infrared
TVAS: transient vibrational absorption spectroscopy
OPA: optical parametric amplifier
CCD: charge-couple device
DHHB: diethylamino hydroxybenzoyl hexyl benzoate
FC: Franck–Condon
ESHT: excited state hydrogen transfer
IC: internal conversion
PES: potential energy surface
CI: conical intersection
MAA: mycosporine-like amino acid
PVA: poly(vinyl) alcohol
DES: diethyl sinapate
VC: VITRO–CORNEUM
CMe: coumaryl Meldrum
SMe: sinapyl Meldrum
CCT: capric/caprylic triglyceride

## References

[ref1] Frederick J. E.; Snell H. E.; Haywood E. K. Solar ultraviolet radiation at the earth’s surface. Photochem. Photobiol. 1989, 50, 443–450. 10.1111/j.1751-1097.1989.tb05548.x.

[ref2] Lucas R.; McMicheal T.; Smith W.; Armstrong B., Solar ultraviolet radiation. Global burden of disease from solar ultraviolet radiation. Environmental Burden of Disease Series, No. 13; World Health Organization: Geneva, Switzerland, 2006.

[ref3] Matsumi Y.; Kawasaki M. Photolysis of atmospheric ozone in the ultraviolet region. Chem. Rev. 2003, 103, 4767–4782. 10.1021/cr0205255. 14664632

[ref4] Taylor H. R.; West S. K.; Rosenthal F. S.; Muñoz B.; Newland H. S.; Abbey H.; Emmett E. A. Effect of ultraviolet radiation on cataract formation. N. Engl. J. Med. 1988, 319, 1429–1433. 10.1056/NEJM198812013192201. 3185661

[ref5] Fisher G. J.; Wang Z. Q.; Datta S. C.; Varani J.; Kang S.; Voorhees J. J. Pathophysiology of premature skin aging induced by ultraviolet light. N. Engl. J. Med. 1997, 337, 1419–28. 10.1056/NEJM199711133372003. 9358139

[ref6] de Gruijl F. R. Skin cancer and solar UV radiation. Eur. J. Cancer 1999, 35, 2003–2009. 10.1016/S0959-8049(99)00283-X. 10711242

[ref7] Dahle J.; Kvam E. Induction of delayed mutations and chromosomal instability in fibroblasts after UVA-, UVB-, and X-radiation. Cancer Res. 2003, 63, 1464–1469. 12670891

[ref8] Gallagher R. P.; Lee T. K. Adverse effects of ultraviolet radiation: a brief review. Prog. Biophys. Mol. Biol. 2006, 92, 119–131. 10.1016/j.pbiomolbio.2006.02.011. 16580054

[ref9] Narayanan D. L.; Saladi R. N.; Fox J. L. Ultraviolet radiation and skin cancer. Int. J. Dermatol. 2010, 49, 978–986. 10.1111/j.1365-4632.2010.04474.x. 20883261

[ref10] Morganroth P. A.; Lim H. W.; Burnett C. T. Ultraviolet Radiation and the Skin:An In-Depth Review. Am. J. Lifestyle Med. 2013, 7, 168–181. 10.1177/1559827612460499.

[ref11] Sinha R. P.; Häder D.-P. UV-induced DNA damage and repair: a review. Photochem. Photobiol. Sci. 2002, 1, 225–236. 10.1039/b201230h. 12661961

[ref12] Rastogi R. P.; Richa; Kumar A.; Tyagi M. B.; Sinha R. P. Molecular mechanisms of ultraviolet radiation-induced DNA damage and repair. J. Nucleic Acids 2010, 2010, 592980 10.4061/2010/592980. 21209706 PMC3010660

[ref13] Bais A. F.; Bernhard G.; McKenzie R. L.; Aucamp P. J.; Young P. J.; Ilyas M.; Jöckel P.; Deushi M. Ozone–climate interactions and effects on solar ultraviolet radiation. Photochem. Photobiol. Sci. 2019, 18, 602–640. 10.1039/C8PP90059K. 30810565

[ref14] Lautenschlager S.; Wulf H. C.; Pittelkow M. R. Photoprotection. Lancet 2007, 370, 528–37. 10.1016/S0140-6736(07)60638-2. 17693182

[ref15] Garone M.; Howard J.; Fabrikant J. A review of common tanning methods. J. Clin. Aesthet. Dermatol. 2015, 8, 43–47. PMC4345932 25741402

[ref16] Cabrera M. I.; Alfano O. M.; Cassano A. E. Absorption and Scattering Coefficients of Titanium Dioxide Particulate Suspensions in Water. J. Phys. Chem. 1996, 100, 20043–20050. 10.1021/jp962095q.

[ref17] Serpone N.; Dondi D.; Albini A. Inorganic and organic UV filters: Their role and efficacy in sunscreens and suncare products. Inorg. Chim. Acta 2007, 360, 794–802. 10.1016/j.ica.2005.12.057.

[ref18] Shaath N. A. Ultraviolet filters. Photochem. Photobiol. Sci. 2010, 9, 464–469. 10.1039/b9pp00174c. 20354639

[ref19] Kockler J.; Oelgemöller M.; Robertson S.; Glass B. D. Photostability of sunscreens. J. Photochem. Photobiol. C, Photochem. Rev. 2012, 13, 91–110. 10.1016/j.jphotochemrev.2011.12.001.

[ref20] Danovaro R.; Bongiorni L.; Corinaldesi C.; Giovannelli D.; Damiani E.; Astolfi P.; Greci L.; Pusceddu A. Sunscreens cause coral bleaching by promoting viral infections. Environ. Health Perspect. 2008, 116, 441–447. 10.1289/ehp.10966. 18414624 PMC2291018

[ref21] Downs C. A.; Kramarsky-Winter E.; Segal R.; Fauth J.; Knutson S.; Bronstein O.; Ciner F. R.; Jeger R.; Lichtenfeld Y.; Woodley C. M.; et al. Toxicopathological Effects of the Sunscreen UV Filter, Oxybenzone (Benzophenone-3), on Coral Planulae and Cultured Primary Cells and Its Environmental Contamination in Hawaii and the U.S. Virgin Islands. Arch. Environ. Contam. Toxicol. 2016, 70, 265–288. 10.1007/s00244-015-0227-7. 26487337

[ref22] Ruszkiewicz J. A.; Pinkas A.; Ferrer B.; Peres T. V.; Tsatsakis A.; Aschner M. Neurotoxic effect of active ingredients in sunscreen products, a contemporary review. Toxicol. Rep. 2017, 4, 245–259. 10.1016/j.toxrep.2017.05.006. 28959646 PMC5615097

[ref23] Gilbert E.; Pirot F.; Bertholle V.; Roussel L.; Falson F.; Padois K. Commonly used UV filter toxicity on biological functions: review of last decade studies. Int. J. Cosmet. Sci. 2013, 35, 208–219. 10.1111/ics.12030. 23237547

[ref24] Fourtanier A.; Moyal D.; Seite S. UVA filters in sun-protection products: regulatory and biological aspects. Photochem. Photobiol. Sci. 2012, 11, 81–89. 10.1039/C1PP05152K. 21904741

[ref25] Gonzalez H.; Tarras-Wahlberg N.; Strömdahl B.; Juzeniene A.; Moan J.; Larkö O.; Rosén A.; Wennberg A.-M. Photostability of commercial sunscreens upon sun exposure and irradiation by ultraviolet lamps. BMC Dermatol. 2007, 7, 1 10.1186/1471-5945-7-1. 17324264 PMC1831786

[ref26] Hojerová J.; Medovcíková A.; Mikula M. Photoprotective efficacy and photostability of fifteen sunscreen products having the same label SPF subjected to natural sunlight. Int. J. Pharm. 2011, 408, 27–38. 10.1016/j.ijpharm.2011.01.040. 21277959

[ref27] Holt E. L.; Stavros V. G. Applications of ultrafast spectroscopy to sunscreen development, from first principles to complex mixtures. Int. Rev. Phys. Chem. 2019, 38, 243–285. 10.1080/0144235X.2019.1663062.

[ref28] Allen J. M.; Gossett C. J.; Allen S. K. Photochemical Formation of Singlet Molecular Oxygen in Illuminated Aqueous Solutions of Several Commercially Available Sunscreen Active Ingredients. Chem. Res. Toxicol. 1996, 9, 605–609. 10.1021/tx950197m. 8728505

[ref29] Kim K.; Park H.; Lim K.-M. Phototoxicity: Its Mechanism and Animal Alternative Test Methods. Toxicol. Res. 2015, 31, 97–104. 10.5487/TR.2015.31.2.097. 26191378 PMC4505355

[ref30] Agnez-Lima L. F.; Melo J. T. A.; Silva A. E.; Oliveira A. H. S.; Timoteo A. R. S.; Lima-Bessa K. M.; Martinez G. R.; Medeiros M. H. G.; Di Mascio P.; Galhardo R. S.; et al. DNA damage by singlet oxygen and cellular protective mechanisms. Mutat. Res. Rev. Mutat. Res. 2012, 751, 15–28. 10.1016/j.mrrev.2011.12.005. 22266568

[ref31] Harada N.; Kataoka M.; Nakanosho M.; Uyama H. Penetration of Singlet Oxygen into Films with Oxygen Permeability Coefficient Close to that of Skin. Photochem. Photobiol. 2021, 97, 971–979. 10.1111/php.13446. 33973245

[ref32] Kao M.-H.; Venkatraman R. K.; Sneha M.; Wilton M.; Orr-Ewing A. J. Influence of the solvent environment on the ultrafast relaxation pathways of a sunscreen molecule diethylamino hydroxybenzoyl hexyl benzoate. J. Phys. Chem. A 2021, 125, 636–645. 10.1021/acs.jpca.0c10313. 33416312

[ref33] Whittock A. L.; Auckloo N.; Cowden A. M.; Turner M. A. P.; Woolley J. M.; Wills M.; Corre C.; Stavros V. G. Exploring the Blueprint of Photoprotection in Mycosporine-like Amino Acids. J. Phys. Chem. Lett. 2021, 12, 3641–3646. 10.1021/acs.jpclett.1c00728. 33826340

[ref34] Abiola T. T.; Rodrigues N. d. N.; Ho C.; Coxon D. J. L.; Horbury M. D.; Toldo J. M.; do Casal M. T.; Rioux B.; Peyrot C.; Mention M. M.; et al. New Generation UV-A Filters: Understanding their Photodynamics on a Human Skin Mimic. J. Phys. Chem. Lett. 2021, 12, 337–344. 10.1021/acs.jpclett.0c03004. 33353308

[ref35] Lakowicz J. R. Principles of fluorescence spectroscopy, 3rd ed.; Springer Science & Business Media: 2013.

[ref36] Baker L. A.; Stavros V. G. Observing and understanding the ultrafast photochemistry in small molecules: applications to sunscreens. Sci. Prog. 2016, 99, 282–311. 10.3184/003685016X14684992086383. 28742490 PMC10365382

[ref37] Berera R.; van Grondelle R.; Kennis J. T. Ultrafast transient absorption spectroscopy: principles and application to photosynthetic systems. Photosynth. Res. 2009, 101, 105–118. 10.1007/s11120-009-9454-y. 19578970 PMC2744833

[ref38] Abiola T. T.; Whittock A. L.; Stavros V. G. Unravelling the Photoprotective Mechanisms of Nature-Inspired Ultraviolet Filters Using Ultrafast Spectroscopy. Molecules 2020, 25, 3945 10.3390/molecules25173945. PMC7504748 32872380

[ref39] Baker L. A.; Greenough S. E.; Stavros V. G. A perspective on the ultrafast photochemistry of solution-phase sunscreen molecules. J. Phys. Chem. Lett. 2016, 7, 4655–4665. 10.1021/acs.jpclett.6b02104. 27791379

[ref40] Liu Y.; Zhao X.; Luo J.; Yang S. Excited-state dynamics of sinapate esters in aqueous solution and polyvinyl alcohol film. J. Lumin. 2019, 206, 469–473. 10.1016/j.jlumin.2018.10.111.

[ref41] Horbury M. D.; Holt E. L.; Mouterde L. M. M.; Balaguer P.; Cebrián J.; Blasco L.; Allais F.; Stavros V. G. Towards symmetry driven and nature inspired UV filter design. Nat. Commun. 2019, 10, 4748 10.1038/s41467-019-12719-z. 31628301 PMC6802189

[ref42] Kukura P.; McCamant D. W.; Mathies R. A. Femtosecond stimulated Raman spectroscopy. Annu. Rev. Phys. Chem. 2007, 58, 461–488. 10.1146/annurev.physchem.58.032806.104456. 17105414

[ref43] Dietze D. R.; Mathies R. A. Femtosecond stimulated Raman spectroscopy. ChemPhysChem 2016, 17, 1224–1251. 10.1002/cphc.201600104. 26919612

[ref44] Schmidt B.; Laimgruber S.; Zinth W.; Gilch P. A broadband Kerr shutter for femtosecond fluorescence spectroscopy. Appl. Phys. B: Laser Opt. 2003, 76, 809–814. 10.1007/s00340-003-1230-7.

[ref45] Sajadi M.; Dobryakov A.; Garbin E.; Ernsting N.; Kovalenko S. Time-resolved fluorescence spectra of cis-stilbene in hexane and acetonitrile. Chem. Phys. Lett. 2010, 489, 44–47. 10.1016/j.cplett.2010.02.034.

[ref46] Cannizzo A.; Bräm O.; Zgrablic G.; Tortschanoff A.; Oskouei A. A.; van Mourik F.; Chergui M. Femtosecond fluorescence upconversion setup with broadband detection in the ultraviolet. Opt. Lett. 2007, 32, 3555–3557. 10.1364/OL.32.003555. 18087540

[ref47] Gerecke M.; Bierhance G.; Gutmann M.; Ernsting N. P.; Rosspeintner A. Femtosecond broadband fluorescence upconversion spectroscopy: Spectral coverage versus efficiency. Rev. Sci. Instrum. 2016, 87, 053115 10.1063/1.4948932. 27250400

[ref48] Stavros V. G.; Verlet J. R. Gas-phase femtosecond particle spectroscopy: a bottom-up approach to nucleotide dynamics. Annu. Rev. Phys. Chem. 2016, 67, 211–232. 10.1146/annurev-physchem-040215-112428. 26980306

[ref49] Staniforth M.; Stavros V. G. Recent advances in experimental techniques to probe fast excited-state dynamics in biological molecules in the gas phase: dynamics in nucleotides, amino acids and beyond. Proc. Math. Phys. Eng. 2013, 469, 20130458 10.1098/rspa.2013.0458. PMC3780818 24204191

[ref50] Baker L. A.; Horbury M. D.; Greenough S. E.; Coulter P. M.; Karsili T. N.; Roberts G. M.; Orr-Ewing A. J.; Ashfold M. N.; Stavros V. G. Probing the ultrafast energy dissipation mechanism of the sunscreen oxybenzone after UVA irradiation. J. Phys. Chem. Lett. 2015, 6, 1363–1368. 10.1021/acs.jpclett.5b00417. 26263136

[ref51] Losantos R.; Sampedro D.; Churio M. S. Photochemistry and photophysics of mycosporine-like amino acids and gadusols, nature’s ultraviolet screens. Pure Appl. Chem. 2015, 87, 979–996. 10.1515/pac-2015-0304.

[ref52] Woolley J. M.; Stavros V. G. Unravelling photoprotection in microbial natural products. Sci. Prog. 2019, 102, 287–303. 10.1177/0036850419877766. 31818205 PMC10424518

[ref53] Bandaranayake W. M. Mycosporines: are they nature’s sunscreens?. Nat. Prod. Rep. 1998, 15, 159–172. 10.1039/a815159y. 9586224

[ref54] Losantos R.; Lamas I.; Montero R.; Longarte A.; Sampedro D. Photophysical characterization of new and efficient synthetic sunscreens. Phys. Chem. Chem. Phys. 2019, 21, 11376–11384. 10.1039/C9CP01267B. 31111130

[ref55] Woolley J. M.; Staniforth M.; Horbury M. D.; Richings G. W.; Wills M.; Stavros V. G. Unravelling the Photoprotection Properties of Mycosporine Amino Acid Motifs. J. Phys. Chem. Lett. 2018, 9, 3043–3048. 10.1021/acs.jpclett.8b00921. 29751729

[ref56] Losantos R.; Churio M. S.; Sampedro D. Computational Exploration of the Photoprotective Potential of Gadusol. ChemistryOpen 2015, 4, 155–160. 10.1002/open.201402125. 25969813 PMC4420587

[ref57] Losantos R.; Funes-Ardoiz I.; Aguilera J.; Herrera-Ceballos E.; García-Iriepa C.; Campos P. J.; Sampedro D. Rational Design and Synthesis of Efficient Sunscreens To Boost the Solar Protection Factor. Angew. Chem., Int. Ed. 2017, 56, 2632–2635. 10.1002/anie.201611627. 28128519

[ref58] Sampedro D. Computational exploration of natural sunscreens. Phys. Chem. Chem. Phys. 2011, 13, 5584–5586. 10.1039/c0cp02901g. 21350786

[ref59] Hatakeyama M.; Koizumi K.; Boero M.; Nobusada K.; Hori H.; Misonou T.; Kobayashi T.; Nakamura S. Unique Structural Relaxations and Molecular Conformations of Porphyra-334 at the Excited State. J. Phys. Chem. B 2019, 123, 7649–7656. 10.1021/acs.jpcb.9b03744. 31430154

[ref60] Koizumi K.; Hatakeyama M.; Boero M.; Nobusada K.; Hori H.; Misonou T.; Nakamura S. How seaweeds release the excess energy from sunlight to surrounding sea water. Phys. Chem. Chem. Phys. 2017, 19, 15745–15753. 10.1039/C7CP02699D. 28604867

[ref61] Conde F. R.; Churio M. S.; Previtali C. M. The photoprotector mechanism of mycosporine-like amino acids. Excited-state properties and photostability of porphyra-334 in aqueous solution. J. Photochem. Photobiol., B 2000, 56, 139–144. 10.1016/S1011-1344(00)00066-X. 11079474

[ref62] Conde F. R.; Churio M. S.; Previtali C. M. The deactivation pathways of the excited-states of the mycosporine-like amino acids shinorine and porphyra-334 in aqueous solution. Photochem. Photobiol. Sci. 2004, 3, 960–967. 10.1039/b405782a. 15480487

[ref63] Singh A.; Čížková M.; Bišová K.; Vítová M. Exploring Mycosporine-Like Amino Acids (MAAs) as Safe and Natural Protective Agents against UV-Induced Skin Damage. Antioxidants 2021, 10, 683 10.3390/antiox10050683. 33925517 PMC8145676

[ref64] Cockell C. S.; Knowland J. Ultraviolet radiation screening compounds. Biol. Rev. Cambridge Philos. Soc. 1999, 74, 311–345. 10.1111/j.1469-185X.1999.tb00189.x. 10466253

[ref65] Rosic N. N. Phylogenetic analysis of genes involved in mycosporine-like amino acid biosynthesis in symbiotic dinoflagellates. Appl. Microbiol. Biotechnol. 2012, 94, 29–37. 10.1007/s00253-012-3925-3. 22361857

[ref66] Woolley J. M.; Losantos R.; Sampedro D.; Stavros V. G. Computational and experimental characterization of novel ultraviolet filters. Phys. Chem. Chem. Phys. 2020, 22, 25390–25395. 10.1039/D0CP04940A. 33141123

[ref67] Urahata S.; Canuto S. Monte Carlo–quantum mechanics study of the UV–visible spectrum of benzophenone in water. Int. J. Quantum Chem. 2000, 80, 1062–1067. 10.1002/1097-461X(2000)80:4/5<1062::AID-QUA55>3.0.CO;2-3.

[ref68] Holt E. L.; Rodrigues N. d. N.; Cebrián J.; Stavros V. G. Determining the photostability of avobenzone in sunscreen formulation models using ultrafast spectroscopy. Phys. Chem. Chem. Phys. 2021, 23, 24439–24448. 10.1039/D1CP03610F. 34694312

[ref69] Dunkelberger A. D.; Kieda R. D.; Marsh B. M.; Crim F. F. Picosecond Dynamics of Avobenzone in Solution. J. Phys. Chem. A 2015, 119, 6155–6161. 10.1021/acs.jpca.5b01641. 25978304

[ref70] Ichimura K.; Yoshizawa M.; Matsuda H.; Okada S.; Ohsugi M. M.; Nakanishi H.; Kobayashi T. Triplet exciton formation due to interaction between singlet excitons in polydiacetylene. J. Chem. Phys. 1993, 99, 7404–7416. 10.1063/1.465721.

[ref71] Sorenson S. A.; Patrow J. G.; Dawlaty J. M. Electronic Dynamics in Natural Iron Pyrite Studied by Broadband Transient Reflection Spectroscopy. J. Phys. Chem. C 2016, 120, 7736–7747. 10.1021/acs.jpcc.5b11036.

